# Mothers’ *Opisthorchis viverrini* infection status and raw fish dish consumption in Lao People’s Democratic Republic: determinants of child infection status

**DOI:** 10.1186/s41182-018-0112-y

**Published:** 2018-08-07

**Authors:** Hitomi Araki, Ken Ing Cherng Ong, Lavy Lorphachan, Pheovaly Soundala, Moritoshi Iwagami, Akira Shibanuma, Bouasy Hongvanthong, Paul T. Brey, Shigeyuki Kano, Masamine Jimba

**Affiliations:** 1 Department of Community and Global Health, Graduate School of Medicine, The University of Tokyo, Tokyo, Japan; 2 Institut Pasteur du Laos, Ministry of Health, Vientiane Capital, Lao People’s Democratic Republic; 3 Department of Tropical Medicine and Malaria, Research Institute, National Center for Global Health and Medicine, Tokyo, Japan; 4 Center of Malariology, Parasitology and Entomology, Ministry of Health, Vientiane Capital, Lao People’s Democratic Republic

**Keywords:** *Opisthorchis viverrini*, Behavior, Children, Mothers, Cross-sectional study

## Abstract

**Background:**

*Opisthorchis viverrini* (Ov) infection is one of the foodborne trematodiases, which is highly endemic in Lao People’s Democratic Republic (PDR). The infection occurs especially when people eat raw fish containing Ov metacercariae. As eating raw fish is a traditional culture in Lao PDR, changing this behavior is difficult. A new approach is necessary to control Ov infection because people easily get re-infected even after taking praziquantel unless they change their behaviors. This study aimed to explore factors associated with Ov infection among children and to identify the existing behaviors and perception that might contribute to the control of Ov infection in Lao PDR. We conducted a cross-sectional study in Yommalath district, Khammouane province, in Lao PDR in August and September 2015. In this cross-sectional study, we used a semi-structured questionnaire and interviewed 348 mothers who had a child aged 5–15 years. We also collected the fecal samples from each mother-child pair and used the Kato-Katz method (three slides/sample) to detect Ov eggs.

**Results:**

Of 284 children, 82.8% were infected with Ov. The children were more likely to be infected with Ov when their mothers were infected with Ov (adjusted odds ratio [AOR] 10.45, 95% confidence interval [CI] 3.13–34.86) or when their mothers liked raw fish dishes (AOR 2.47, 95% CI 1.07–5.69). Even though most mothers are primarily in charge of cooking family meals, fathers were also involved in the preparation of raw fish dishes.

**Conclusion:**

This study suggests that a new approach to control Ov infection should target families or communities, rather than children only. Cooking or food preparation behaviors should be investigated in more depth.

## Background

*Opisthorchis viverrini* (Ov) infection is one of the neglected diseases categorized as a foodborne trematode infection. It is endemic in countries along the Mekong River basin, especially in Lao People’s Democratic Republic (PDR) and northeast Thailand, where 10 million people are infected with Ov [1]. In Lao PDR, almost half of the population is infected with Ov foodborne trematodiases, which often results in disability at the later stage of an infected person’s life [2, 3]. Ov infection is among the risk factors of bile duct cancer (cholangiocarcinoma) [4].

In those Ov-endemic areas, it was found that people have been eating raw fish dishes as part of their traditional culture [5, 6]. In the life cycle of Ov, the intermediate host is the commonly eaten freshwater fish [7]. Thus, the habit of eating raw fish was among the reasons why people developed Ov infection [8]. The common preventive measure is to avoid eating raw fish and to cook the fish properly; however, this behavioral change is difficult because eating raw fish is a practice deeply rooted in their culture [9]. In addition, people in Lao PDR like raw fish dishes, such as fish salad (*koi pa*) [5].

To control Ov infection, mass drug administration (MDA) of praziquantel was conducted in many endemic areas. In Lao PDR, MDA was conducted in six provinces with high (> 20%) Ov prevalence, namely, Attapeu, Bolikhamxay, Champasak, Khammouane, Saravan, and Savannakhet. In Khammouane province, MDA has been conducted since 2011, and praziquantel was provided to the risk group aged 5–60 years every October through the support of a donor agency such as the World Health Organization (WHO) (personal communication with a WHO officer in Lao PDR).

Even though praziquantel is widely used to treat Ov infection, people would be infected repeatedly unless they change their behaviors. In northeast Thailand, the Ov re-infection rates 6 and 12 months after the treatment were 4.5% and 6.2%, respectively [10]. Ov re-infection is likely to occur as fast as 2 months after treatment with praziquantel [11].

To control Ov infection, the importance of community-based intervention has been emphasized. However, household-level studies are limited, particularly in Lao PDR. This study aimed to target the population with lower risk of Ov infection, i.e., children and women. Generally, children’s eating behaviors depend on the households’ eating habits, and mothers are usually in charge of preparing food for them. We assumed that the children’s risk of getting infected with Ov would increase if household members (e.g., mothers) like raw fish. Even though mothers like raw fish dishes, they may not let their children eat raw fish if they think it is risky. In this case, their children may not be at risk of developing Ov infection. People also eat raw fish dishes while drinking alcohol especially among adult males in Ov-endemic areas. Thus, we assumed mothers’ practices would influence children’s Ov status more than those of fathers. In Khammouane province, children [12] or women [13] were less likely to be infected with Ov. Nevertheless, a nationwide survey among primary school children showed that the prevalence of Ov infection in Khammouane province was 33.2%, which was the highest among all the provinces [14]. In Savannakhet province, a study on school children’s Ov infection found many associated factors, such as frequency of eating raw fish [7]. In this study, we focused more on the behaviors in households, including food preparation. We conducted this study to explore factors associated with Ov infection among children and identify the existing behaviors and perception that might contribute to control Ov infection in Lao PDR.

## Methods

### Study area

We conducted this cross-sectional study in Khammouane province in August and September 2015. This province is located in the central part of Lao PDR, and we purposely selected it as it is one of the Ov-endemic provinces in Lao PDR and relatively few studies were conducted there compared to the southern provinces [3, 13, 15–20].

Of the nine districts in Khammouane province, we purposively selected Yommalath district as it is one of the most Ov-endemic districts (personal communication with Center of Malariology, Parasitology and Entomology). This district is located about 45 km east from Thakhek, the capital city of Khammouane province. Yommalath district is accessible from Thakhek by a 1-h travel by car via Route 13. Yommalath has a district hospital with 15 beds, 7 health centers, and 53 schools. Among the schools, only 37 (69%) have toilets. The main occupation of the residents is farming. The average annual income per person is $1235 (personal communication with the district health officer).

Of all 46 villages in the district, 16 had the data of Ov prevalence in 2012 (unpublished data from Yommalath district health office). We divided those 16 villages into two groups, four villages (Ov prevalence ≥ 12%) and 12 villages (Ov prevalence < 12%). We randomly selected two and three villages using lottery method.

### Sample size calculation

We calculated the sample size using OpenEpi version 3.03a, setting the confidence level at 95% and power at 90%. The minimum sample size was 310. Considering the 15% missing data, the required sample size was 365.

### Preparation of the questionnaire

We prepared the questionnaire for the face-to-face interview in English, which was adapted from previous studies [7, 21, 22]. The questionnaire included questions on sociodemographic status, eating behavior, fishing behavior, raw food consumption, knowledge of Ov, perception on feeding raw fish, drugs (including traditional medicine) usually taken, and food eaten for health. A Lao researcher translated the questionnaire into Lao, and another Lao researcher translated it back into English to check the consistency. We pretested it in a village in Paknguem district, Vientiane capital. After the pretest, we eliminated or modified difficult or irrelevant questions.

We modified a wealth index adapted from the questionnaire used in Lao Social Indicator Survey in 2011–2012, asking about possession of household assets and animals [23]. For its calculation, all 28 items were recorded as binary variables: 0 (do not have household assets and animals) or 1 (have household assets and animals). We calculated each individual wealth score and divided the scores by household size. Then, we divided the weighted scores into quintiles from the poorest to the richest.

### Face-to-face interview and stool examination

Before the study, we informed a village leader or a village health worker about this research and asked them to inform all the mothers in the village who had a child aged 5–15 years. We also asked the mothers to come to a meeting place or a temple in the village for the interview. During the interview, mothers freely selected one child aged 5–15 years, depending on their preferences, to provide a fecal sample. We interviewed all the mothers who came to the place. The interview took about 30–50 min per person. We recorded the answers in Lao languages and translated them to English for analysis. We collected fecal samples from each mother-child pair and stored them at 4 °C until examination. We examined 25 mg of sample using triple Kato-Katz thick smear method and considered Ov positive if at least one of three slides had Ov eggs.

### Data management and statistical analysis

We used Epi Info 7 to enter new data. After exporting the data into Excel 2013, we checked the data twice to avoid data entry errors. We used Stata version 13.0 (College Station, Texas, USA) for quantitative data analysis and categorized qualitative data such as answers to open-ended questions prior to analysis. A *p* value less than 0.05 was considered significant. *χ*^2^ test and Fisher’s exact test were used to compare categorical variables, and *t* test was used to compare continuous variables. We also performed a multivariate logistic regression analysis to identify the associated factors.

## Results

In total, 348 mothers participated in the interview. Of those, we used 338 data for descriptive analysis after excluding 10 data due to missing fecal samples or children’s age younger than 5. After conducting the descriptive analysis, we dropped 54 data, which had answers “do not know” to the questions asking about ethnicity, mothers’ education status, or religion. Therefore, we used 284 data for multiple logistic regression analysis. The overall Ov infection rate was 87.9% (*n* = 676; both mothers and children). The Ov infection rate among children (*n* = 338) was 82.8%, whereas that of mothers (*n* = 338) was 92.9%.

We summarized the sociodemographic characteristics in Table 1. The mean household size was 5.5 (standard deviation (SD) 1.7) persons. The mean age of the mothers and their children were 37.1 (SD 9.0) years and 10.0 (SD 3.4) years, respectively. The major ethnic group was Lao-Tai (80.3%), followed by Mon-Khmer (19.7%). Among the mothers, 37.0% were illiterate and 52.5% had primary school education. With regard to the use of toilet facility, 43.0% did not use any. Of 284 children, 235 (82.8%) were positive for Ov (Table 2). Mothers’ preference for raw fish (adjusted odds ratio (AOR) 2.47, 95% confidence interval (CI) 1.07–5.69) and mothers’ Ov status (AOR 10.45, 95% CI 3.13–34.86) were positively associated with children’s Ov infection (Table 3).

**Table 1:** Characteristics of the mothers and comparison by children’s Ov status (*n* = 284)

	*n* (%)		Children’s Ov status				*p* value
			Negative, *n* (%)		Positive, *n* (%)		
Children’s sex							
Male	158	(55.6)	23	(14.6)	135	(85.4)	0.178
Female	126	(44.4)	26	(20.6)	100	(79.4)	
Ethnicity							
Lao-Tai	228	(80.3)	42	(18.4)	186	(81.6)	0.331^†^
Mon-Khmer	56	(19.7)	7	(12.5)	49	(87.5)	
Education level of mother							
Illiterate/no schooling	105	(37.0)	16	(15.2)	89	(84.8)	0.168^†^
Primary	149	(52.5)	24	(16.1)	125	(83.9)	
Secondary	30	(10.6)	9	(30.0)	21	(70.0)	
Marital status							
Married	266	(93.7)	48	(18.1)	218	(82.0)	0.346^†^
Widowed	11	(3.9)	0	(0.0)	11	(100.0)	
Divorced	7	(2.5)	1	(14.3)	6	(85.7)	
Religion							
Buddhist	186	(65.5)	38	(20.4)	148	(79.6)	0.005
Animist	98	(34.5)	11	(11.2)	87	(88.8)	
Occupation							
Rice farmer	276	(97.2)	45	(16.3)	231	(83.7)	0.032^†^
Other*	8	(2.8)	4	(50.0)	4	(50.0)	
Toilet mainly used							
No facility	122	(43.0)	14	(11.5)	108	(88.5)	0.007
Pit latrine	111	(39.1)	19	(17.1)	92	(82.9)	
Flush	51	(18.0)	16	(31.4)	35	(68.6)	

Total may not become 100% due to rounding off

^†^Fisher’s exact test

*Teacher/civil servant (*n* = 4), no occupation (*n* = 3), trade/business (*n* = 1)

**Table 2:** Ov-infected children by age and sex (*n* = 284)

	Total (*n* = 284)		Ov positive						*p* value
			Total (*n* = 284)		Male (*n* = 158)		Female (*n* = 126)		
Age of children, mean (SD)	10.0	(3.4)	10.2	(3.4)	9.7	(3.3)	10.8	(3.3)	0.032*
Age group, *n* (%)									0.345**
5 years	33	(11.6)	25	(75.8)	16	(80.0)	9	(69.2)	
6–10 years	126	(44.4)	101	(80.2)	63	(84.0)	38	(74.5)	
11–14 years	87	(30.6)	75	(86.2)	40	(88.9)	35	(83.3)	
15 years	38	(13.4)	34	(89.5)	16	(88.9)	18	(90.0)	
Total	284	(100.0)	235	(82.8)	135	(85.4)	100	(79.4)	

*Comparing the mean age of children by children’s Ov status

**Comparing the children’s Ov status by age group

**Table 3:** Factors associated with children’s Ov status (*n* = 284)

	OR	95% CI	*p* value	AOR	95% CI	*p* value
Children’s sex						
Male (ref)						
Female	0.76	(0.43–1.34)	0.345	0.47	(0.21–1.05)	0.065
Age of child	1.11	(1.01–1.22)	0.034	1.08	(0.94–1.24)	0.258
Age of mother	1.06	(1.02–1.10)	0.004	1.05	(0.99–1.11)	0.083
Ethnicity						
Lao-Tai (ref)						
Mon-Khmer	1.58	(0.67–3.73)	0.297	0.64	(0.05–7.54)	0.724
Education status of mother						
No schooling/illiterate (ref)						
Primary	0.94	(0.47–1.86)	0.851	2.05	(0.84–5.03)	0.115
Secondary	0.42	(0.16–1.08)	0.072	1.31	(0.38–4.48)	0.668
Wealth index						
Poorest (ref)						
Poorer	0.48	(0.13–1.70)	0.255	1.16	(0.25–5.51)	0.849
Middle	0.33	(0.10–1.08)	0.066	0.73	(0.17–3.20)	0.675
Richer	0.60	(0.17–2.11)	0.424	1.37	(0.28–6.80)	0.701
Richest	0.22	(0.07–0.72)	0.012	0.73	(0.13–3.96)	0.715
Main fuel						
Wood (ref)						
Charcoal/coal/lignite	0.39	(0.21–0.73)	0.003	0.58	(0.23–1.45)	0.242
Main toilet						
No facility (ref)						
Pit latrine	0.63	(0.30–1.32)	0.220	1.12	(0.40–3.10)	0.828
Flush	0.28	(0.13–0.64)	0.002	0.66	(0.20–2.16)	0.493
Mother likes raw fish						
No (ref)						
Yes	2.95	(1.47–5.95)	0.002	2.47	(1.07–5.69)	0.033
Mothers’ Ov status						
Negative (ref)						
Positive	5.77	(2.25–14.77)	< 0.001	10.45	(3.13–34.86)	< 0.001

**Fig. 1:**
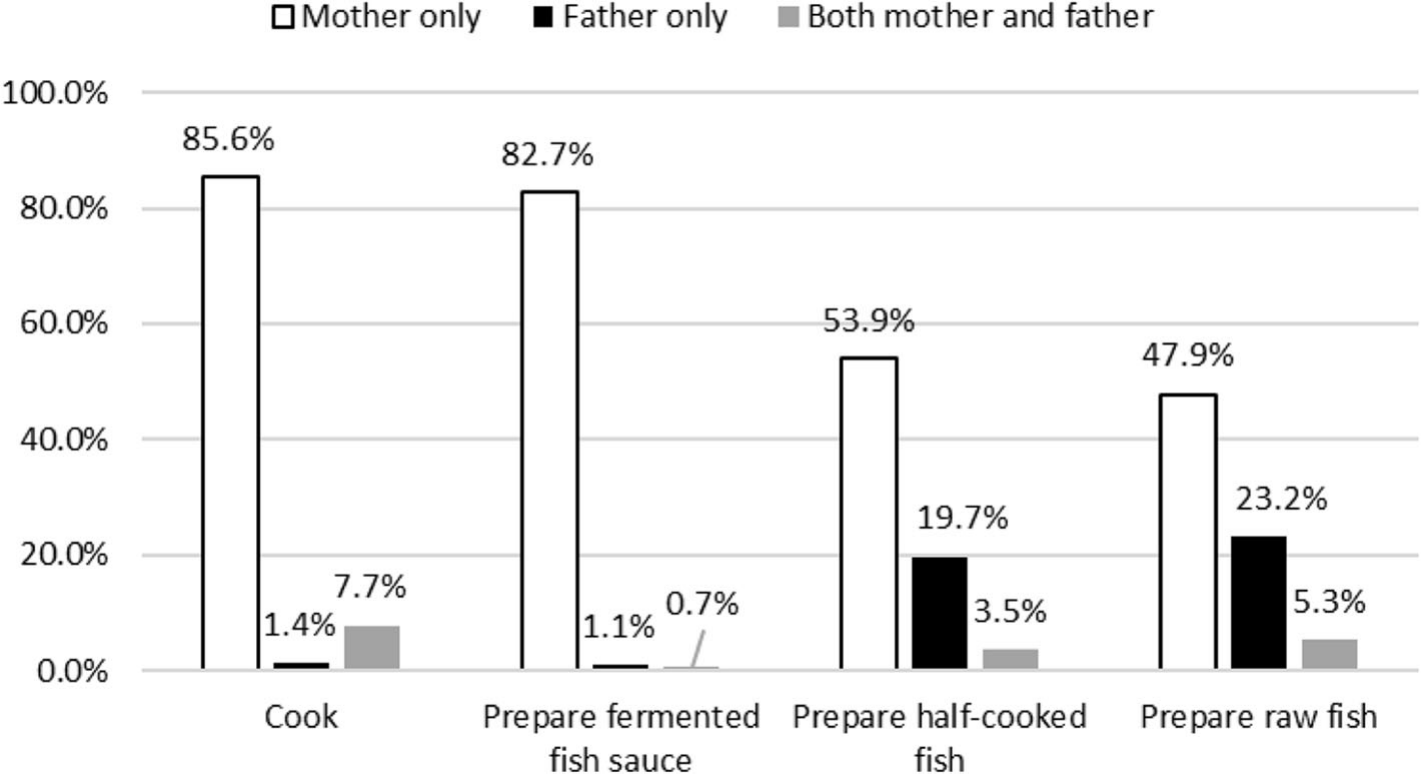
Parents’ cooking responsibility (*n* = 284)

As for parents’ cooking responsibility, fathers were more likely to be in charge of preparing half-cooked or raw fish dishes than perform the usual cooking method (Fig. 1) although mothers were more involved in usual cooking.

**Fig. 2:**
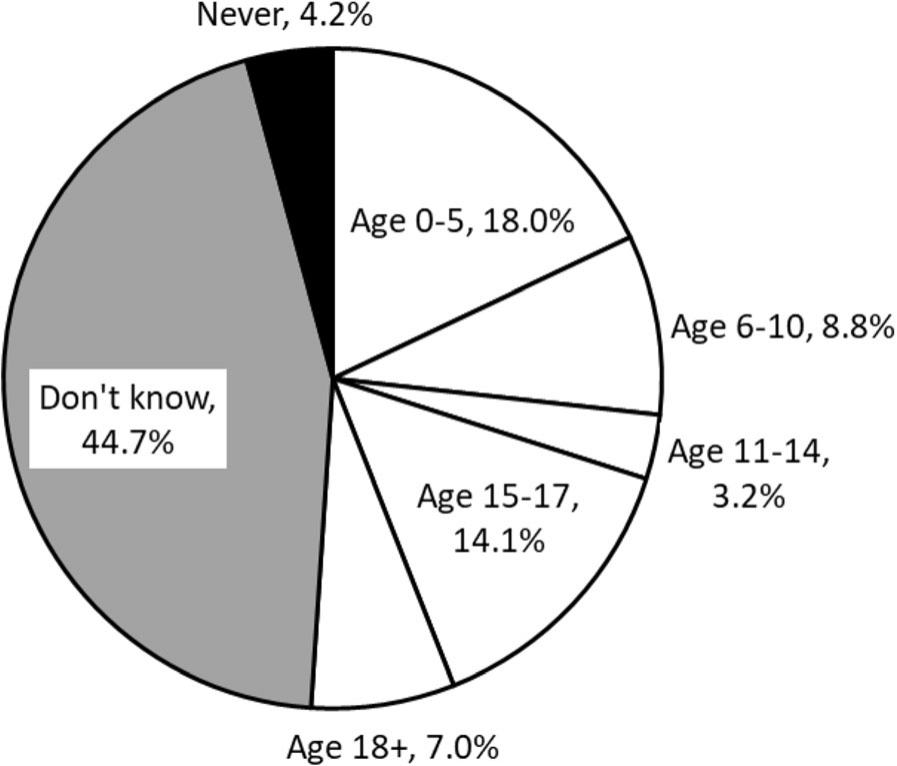
Mothers’ perception on the minimum age to start feeding or eating raw fish (*n* = 284)

**Fig. 3:**
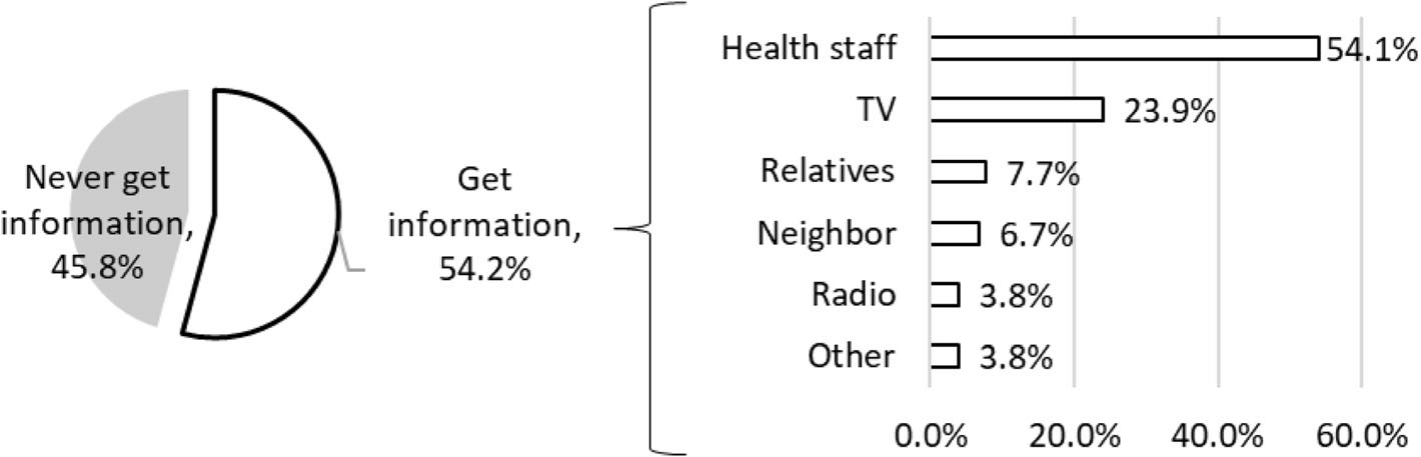
Information source of Ov (*n* = 284, multiple answers except for never get information)

We found a variation in mothers’ perception on the minimum age to start feeding or eating raw fish. Among all mothers, 44.7% answered that they did not know when to start feeding their children or allowing them to eat raw fish, and 4.2% answered that they would never feed children raw fish (Fig. 2). The remaining mothers perceived that the minimum age to start feeding their children or allowing them to eat raw fish was from 0 to 40 years old. Of 284 mothers, 45.8% have no knowledge about Ov infection (Fig. 3).

## Discussion

Children’s Ov infection was associated with mothers’ Ov infection and their preference for raw fish dishes. While all of the mothers mentioned the risk for children to eat raw fish, only a few mothers mentioned Ov infection. Mothers were mainly responsible for cooking, but fathers were also involved in it, especially in the preparation of raw fish dishes.

Children were more likely to be infected with Ov if their mothers were infected with Ov (Table 2). This finding is new and would raise three possibilities: (i) Sharing the same raw fish dishes in a family may cause both mother and child Ov infection, (ii) the defecation site and the fishing site of a household may be the same, and (iii) infected raw fish may contaminate utensils during food preparation or hands while eating.

Although some parents prepare raw fish dishes for adults and cooked fish for children separately, the parents usually eat both dishes by hand or with spoon. Thus, even if children eat only cooked fish, they may get infected due to contamination. However, no study has focused on the association between Ov infection and safe food preparation, especially on intra-household Ov contamination. Thus, another biological study is necessary to examine Ov metacercariae remaining on utensils. Ov metacercariae are distributed in various body parts of a fish, namely in the muscles (58%), fins (28%), heads (13%), and visceral organs (2%) [17]. Metacercariae were also observed in gray spots of cyprinoid fish scales [23]. In our study area, washing chopping boards with sponge and detergent was an uncommon behavior, although most of the households had them for dish washing. The mothers usually scratched chopping boards using a knife with water. In some houses, we observed fish scales remaining on the chopping boards. Therefore, chopping boards may be a potential source of contamination. Thus, further research is needed to examine this possibility. The potential of contamination due to metacercariae on surface of cooking utensils was also considered in previous studies on the similar liver fluke, *Clonorchis sinensis*, in Vietnam [24] and China [25]. In the study area, about 70% of the households had a refrigerator, which usually contains a freezer section. Although the biological evidence is still lacking, freezing fish might be helpful in reducing the risk of Ov infection. These behavioral changes might be more feasible and culturally more acceptable than banning the consumption of raw fish [26].

Mothers’ preference for raw fish was associated with children’s Ov infection. However, fathers’ preference for raw fish was not associated. Previously, parents’ preference for raw fish was found to be associated with children’s Ov infection in Lao PDR [17]. This result might be due to two different units at risk of eating raw fish dishes, that is, the household and the adult males’ community. This hypothesis may also be supported by our result that fathers also prepared half-cooked or raw fish dishes but mothers were mostly responsible for general cooking (Fig. 1). In Vientiane province, women were usually in charge of cooking in households, whereas many men prepared raw fish dishes by themselves [27]. In Saravane province, eight women confirmed that women mostly prepared raw fish dishes in the focus group discussions and that adult men tended to eat more raw fish dishes [28]. In our study, some mothers reported that they did not know how to prepare raw fish dishes but their husband knew and prepared them. Therefore, when intervention on food preparation is planned, both mothers and fathers should be involved, targeting the household and the adult males’ community, respectively. The intervention on mothers may contribute in reducing the risk of Ov infection among their children.

With regard to the perception on minimum age to start feeding or eating raw fish, our result was slightly different from those of previous studies conducted in Saravane district, Saravane province. In a qualitative study, eating raw fish was usually allowed in children aged 14 years old [5]. In another cross-sectional study, nearly half of the heads of households allowed their children to consume raw fish once they are able to feed themselves, mostly at age 3 and even at age 2 [28]. Some of the mothers in our study told us that their children had eaten raw fish dishes but nothing happened. As most of the Ov-infected cases are asymptomatic and stool examination is rarely performed in the study area, it is difficult for local people to know whether they are infected with Ov. Local mothers may consider that they can start allowing their children to eat raw fish dishes based on their symptoms. If mothers are well informed about Ov infection, it might help prevent young children from early exposure to raw fish consumption.

The Ov infection rate was 87.9% (*n* = 676; mothers and children), 92.9% among mothers (*n* = 338) and 82.8% among children (*n* = 338). This rate was higher than that among villagers in Thakhek district (54.8%, *n* = 237), the capital of the province [13]. It was also considerably higher than the infection rate reported in southern endemic provinces in Lao PDR [1, 29]. This may be due to the different number of stool samples examined. The study on household members (*n* = 574) in Saravane district, Saravane province, showed an 88.7% Ov infection prevalence using two stool samples examined using Kato-Katz method [28]. In our method, examining three slides might have increased the sensitivity. We were not able to differentiate the eggs of minute intestinal flukes from Ov eggs because they look very similar. Therefore, the Ov infection rate might be overestimated.

This study had some limitations that should be considered. First, the children were not interviewed in this study, which may have enabled us to get more information especially on children’s preference and knowledge about Ov infection or potential factors associated with children’s Ov infection. Second, we asked each mother to select one of her children without any criteria other than age. Thus, the mothers may have selected healthier children. Third, regarding raw fish consumption, underreporting bias may exist because all the mothers were aware of more or less the health risks of feeding raw fish. Despite the limitations, this study provides the perspectives and indicates the role of families, which may potentially contribute to a new approach to control Ov infection at family or community level.

## Conclusion

We found two risk factors associated with children’s Ov infection: (1) mothers’ preference for raw fish dishes and (2) the incidence of Ov infection among mothers. Although mothers were usually in charge of cooking family meals, fathers were also involved especially in preparing raw fish dishes. Hence, we need to further investigate on the possibility of intra-household Ov contamination when the members of the household have the habit of eating raw fish dishes, preserving fish, and preparing raw fish dishes, which might lead to a new approach to control Ov infection. Hence, future interventions, for example MDA, should involve families rather than children only.

## Abbreviations

AOR: Adjusted odds ratio
Lao PDR: Lao People’s Democratic Republic
MDA: Mass drug administration
Ov: *Opisthorchis viverrini*
SD: Standard deviation
